# Involving Animal Models in Uterine Transplantation

**DOI:** 10.3389/fsurg.2022.830826

**Published:** 2022-02-23

**Authors:** Angeline Favre-Inhofer, Marie Carbonnel, Johanna Domert, Nathalie Cornet, Sylvie Chastant, Raphaël Coscas, François Vialard, Valérie Gelin, Laurent Galio, Christophe Richard, Héla Trabelsi, Olivier Sandra, Dominique de Ziegler, Pascale Chavatte-Palmer, Jean-Marc Ayoubi

**Affiliations:** ^1^Department of Gynaecology and Obstetrics, Foch Hospital, Suresnes, France; ^2^Université Paris-Saclay, UVSQ, INRAE, BREED, Jouy-en-Josas, France; ^3^NeoCare, ENVT, Université de Toulouse, Toulouse, France; ^4^Department of Vascular Surgery, Ambroise Paré University Hospital, AP-HP, Boulogne-Billancourt, France; ^5^UMR 1018, Inserm-Paris11 - CESP, Versailles Saint-Quentin-en-Yvelines University, Paris-Saclay University, Boulogne-Billancourt, France; ^6^École Nationale Vétérinaire d'Alfort, BREED, Maisons-Alfort, France; ^7^Département de Génétique, Laboratoire de Biologie Médicale, CHI de Poissy-St Germain en Laye, Poissy, France

**Keywords:** uterus, transplantation, surgery, sheep, animal, animal models

## Abstract

**Background:**

Absolute uterine factor infertility affects 0. 2% women of childbearing age around the world. Uterine transplantation (UTx) is a promising solution for many of them since the first birth from UTx was described by the Swedish team in 2014. The success of Utx in humans has become possible after a systematic and meticulous approach involving years of research on animal models. To date, more than 80 UTx procedures have been performed worldwide and 30 children were born.

**Material and Method:**

This review summarizes the research preparation conducted in animals before beginning UTx in humans. It focuses on the advantages and limits of each animal model, their place in surgical training, and current contribution in research to improve UTx successes in humans. The different steps in the process of UTx have been analyzed, such as imaging, surgery, ischemia-reperfusion effects, rejection markers, immunosuppressive treatment, and pregnancy.

**Conclusion:**

Animal models have played an essential role in the implementation of UTx, which is a highly complex procedure. While respecting the 3R requirements (replacement, refinement, and reduction), the surgical training using large animal models, such as notably ewes remain irreplaceable for teams wishing to initiate a UTx program. Furthermore, animal models are still mandatory in current research to improve the success rates of UTx in humans as well as to reduce the morbidity associated with this experimental infertility treatment.

## Introduction: The Swedish Model

Absolute Uterine factor infertility affects one in 500 women of childbearing age (1). These women's options for bearing a child are adoption, surrogate motherhood, or uterine transplantation (UTx). The first livebirth after UTx in women occurred in 2014 in Sweden (2), as part of the first world UTx series. Among nine UTx performed, seven were successful and eight healthy children were born (3). This high rate of success resulted from a long and meticulous preparation.

Using an animal model is a preliminary and mandatory step to any surgical innovation as mentioned in Moore's criteria and the idea, development, exploration, assessment, long-term study (IDEAL) concept (4, 5). In 2009, the International Federation of Gynecology and Obstetrics (FIGO) recommended preliminary studies in several animal models, such as non-human primate prior to undertaking UTx (6).

The Swedish team, led by Mats Brännström, studied all aspects of UTx in numerous animal models during more than a decade before performing their first trial in humans. They worked initially on mice and rats and reported the first livebirth after UTx in syngeneic mice in 2003 (7) and after allotransplantation in 2010 (8). They subsequently worked on larger animal models, such as sows since 2004 (9) and ewes since 2005 leading to the first livebirth after auto-transplantation reported in 2010 (10). Ultimately, this team worked on baboons in 2008. Based on the experience gained using animal models, Brännström's designed a human protocol. Despite accomplishments achieved in humans, they still train regularly on sheep for practicing surgery because it is the model closest to humans.

The challenge is to stay ahead in this high-level set fertility performances while respecting the ethical aspects of animal research, represented among others by the 3R system, such as replacement, refinement, and reduction (11).

We searched articles in English in PubMed using the Keywords: “uterus,” “uterine,” “transplant,” “transplantation,” and subsequently AND mice, AND rat, AND pig, AND sheep, AND ewe, AND macaque, and AND baboon. We excluded all reviews, articles on other topics, articles not in English, articles published before 2000. We excluded some animal models because they had limited impact (rabbits, dogs, and cats).

In this review, we describe the strengths and weaknesses of the different animal models. In addition, we explain their respective contribution in different aspects of UTx notably, in terms of surgical training and research (imaging, surgery, ischemia-reperfusion, rejection, immunosuppressive treatment, and gestations). Finally, we look at some possible future research projects based on animal models.

## Strengths and Weaknesses of the Various Animal Models

Advantages and disadvantages of different animal models for UTx are described in Table 1.

**Table 1:** Advantages and disadvantages of different animal models for UTx.

**Species**		**Advantages**	**Disadvantages**
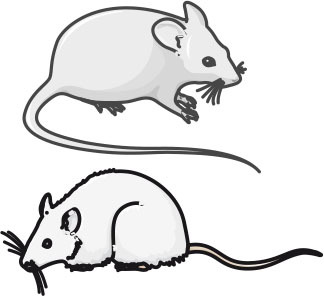		Availability Syngeneic model Ethical acceptability Cost Fertility Short pregnancy (19–20 days)	Size Phylogenetic difference
			
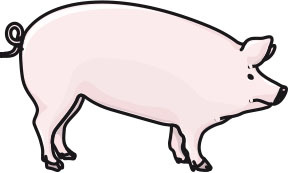		Size Similar uterine and pelvic vascular anatomy Availability Fertility Longer pregnancy (1/3 of human pregnancy)	Pelvic and uterine anatomy Small size of vessels Phylogenetic distance Cost Limited ethical acceptability Phylocotous
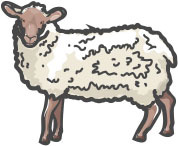		Size Pelvic anatomy Vessel size Availability Longer pregnancy (1/2 of human pregnancy)	Venous uterine drainage Postoperative recovery Phylogenetic distance Cost Limited ethical acceptability Only one or two offspring in general
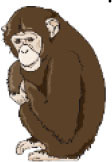	Macaque	Pelvic anatomy Uterine anatomy	Size Small size of vessels Angled cervix Limited availability Cost Very limited ethical acceptability Limited fertility Longer pregnancy (around 180 days) Only one or two offspring in general
	Baboon	Size Pelvic anatomy Uterine anatomy Linear cervix Phylogenetically close to human	Availability Cost Ethics limitations Very limited ethical acceptability Longer pregnancy (around 180 days)

The first successful UTx using an animal model was conducted in rodents. This species is easily available and has perfectly known physiology, immunology, reproduction, and genetics. Moreover, practicing UTx with a syngeneic model permits to avoid immune reaction (12, 13). Gestation period is short allowing an easy and repeated study. The cost of the procedure is relatively low, and studies can be made on numerous animals. The main drawback is its anatomical size. Indeed, for the surgical training, small animals are unfortunately inappropriate because of the size difference with humans. This is especially the case when vessel dissection and anastomosis are performed. It is therefore essential to practice surgery on large animals.

However, choosing the right large-animal model, however, is difficult. Studies were led on pigs (9, 14), which have similar vascular anatomy. Unfortunately, the pelvic anatomy is very different from that of humans. The uterus of the pig is composed of two long horns of 1 m each, making hysterectomy and anastomoses difficult and different from humans. In spite of these differences, some research was conducted: a heterotopic allogeneic model in mini-pigs (15) usable for basic research and an auto-transplantation model using the ovarian vein (16). Gestation is about one-third of the humans (110 days) with a large litter size. For most teams, the pig model can be used for research, but is not the right animal model for practicing transplantation surgery in preparation for the human UTx.

**Figure 1:**
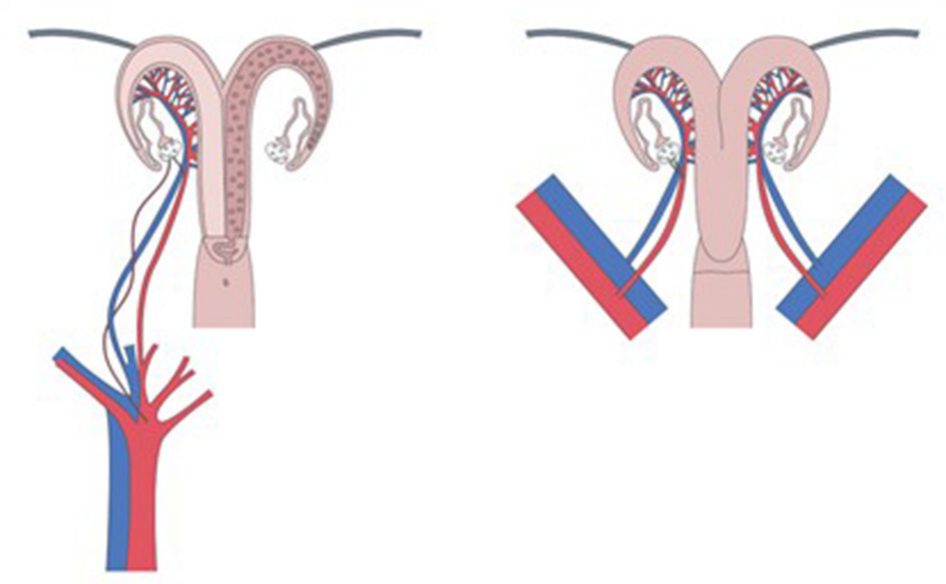
Uterus autotransplantation in sheep.

Ewes represent a better model than pigs because of their similarity with humans in terms of body size, pelvic anatomy, and uterine vessel size (17). It is the best animal model for surgical training in UTX (18). Uterine arterial vascularization is very similar to that of humans, but the venous drainage is quite different. There are two utero-ovarian veins in the ewe while there are two uterine veins and two ovarian veins in women (19). Furthermore, the uterus is bicornuate in ewes (Figure 1). The difficulty lies in the risk of ileus blockage and difficulty of recovering the rumination process. Sheep are phylogenetically relatively far from humans in terms of immunosuppression processes, making research in this field difficult. The gestation time is nearly half of that observed in human (145 days), with 1–2 fetuses of human size. This facilitates studying pregnancies and the development of lambs.

Non-human primates appear as a very good model for UTx notably, because anatomy and more specifically, vascular anatomy is very similar to that of humans. Cynomolgus and rhesus macaques (20) were used as UTx model but surgery is difficult because of the small size of macaques and their vessels. Another limitation is the reduced fertility with monthly ovulation, single and long pregnancy. Moreover, the cervix is angled, which makes cervical biopsies and embryo transfers problematic. For these reasons, trials were performed in baboons (21), that are bigger but still half the size of human beings. Their cervices are also linear. Nevertheless, even if non-human primates offer a model of choice because of their anatomical and phylogenetic similarities to humans, their use is ethically very difficult, and their cost remains high. The availability of these species for transplantation research is therefore very limited. Thus, although these were necessary before initiating the world first UTx cases, the primate model is not appropriate any more for current research or training in view of UTx.

## Surgical Training

Starting a new UTx program is a complex multidisciplinary process. UTx is more complex than other major live donor transplantation procedures.

Live donor surgery is a far more extensive procedure than a simple hysterectomy. The uterine vessels must be preserved, which enhances the difficulty and can lead to major complications. The dissection of uterine veins is complex due to their proximity to the ureters and the number of small venous branches. A complete ureterolysis and removal of a patch of the internal iliac vessels is also necessary. Moreover, substantial portions of the uterine ligaments, an extensive sheet of bladder peritoneum, and part of the vagina are also needed for the anastomosis with the recipient. The duration of the live donor surgery—removal of the uterus—in the hands of the high-skilled Swedish team was 10–13 h (22). The introduction of robotic surgery was shown to improve the dissection and postoperative recovery, though the surgery duration was not reduced (23).

**Figure 2:**
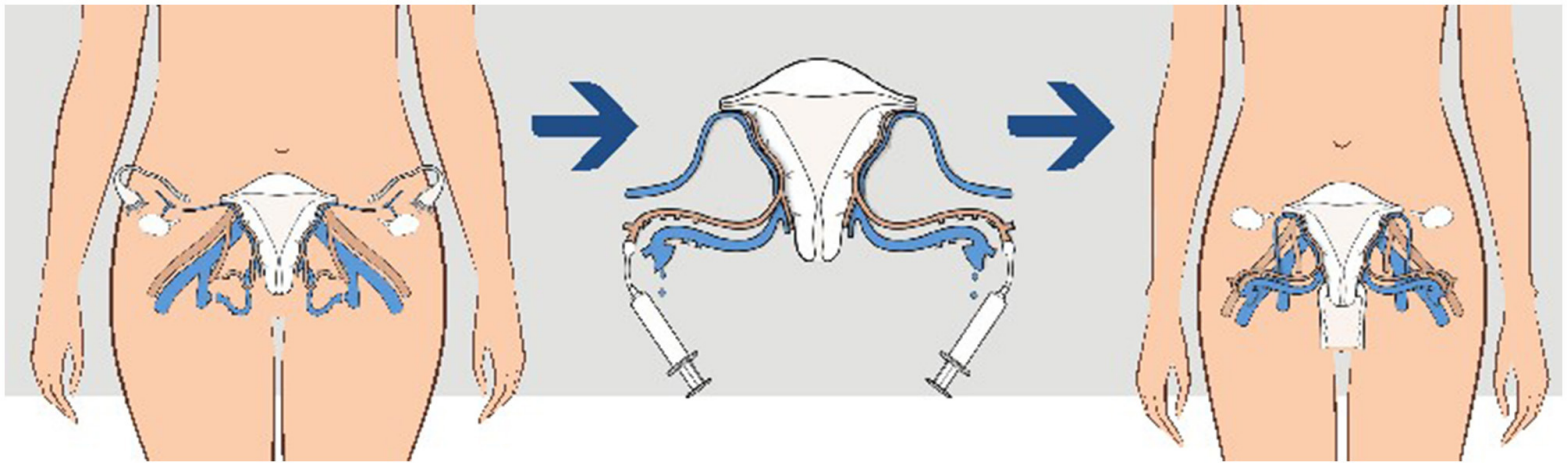
Steps of uterine transplantation (UTx) in human.

The recipient's surgical operation is performed by laparotomy. The first step is the dissection of the vaginal vault from the bladder and rectum. The external iliac vessels are then exposed, and the uterine graft is placed in the orthotopic position with end–to-side anastomoses between uterine vessels and iliac extern vessels. The average duration of surgery for the recipient lasts close to 5 h (22). Microsurgery skills using 7.0 sutures are needed for vascular anastomoses. Transplantectomy is necessary in 20% of cases mainly due to thrombosis (3). The different steps are represented in Figure 2.

A meticulous preparation and optimal settings are necessary components for the responsible introduction of UTx. Moreover, prior surgical training on animal models is seminal for success.

**Figure 3:**
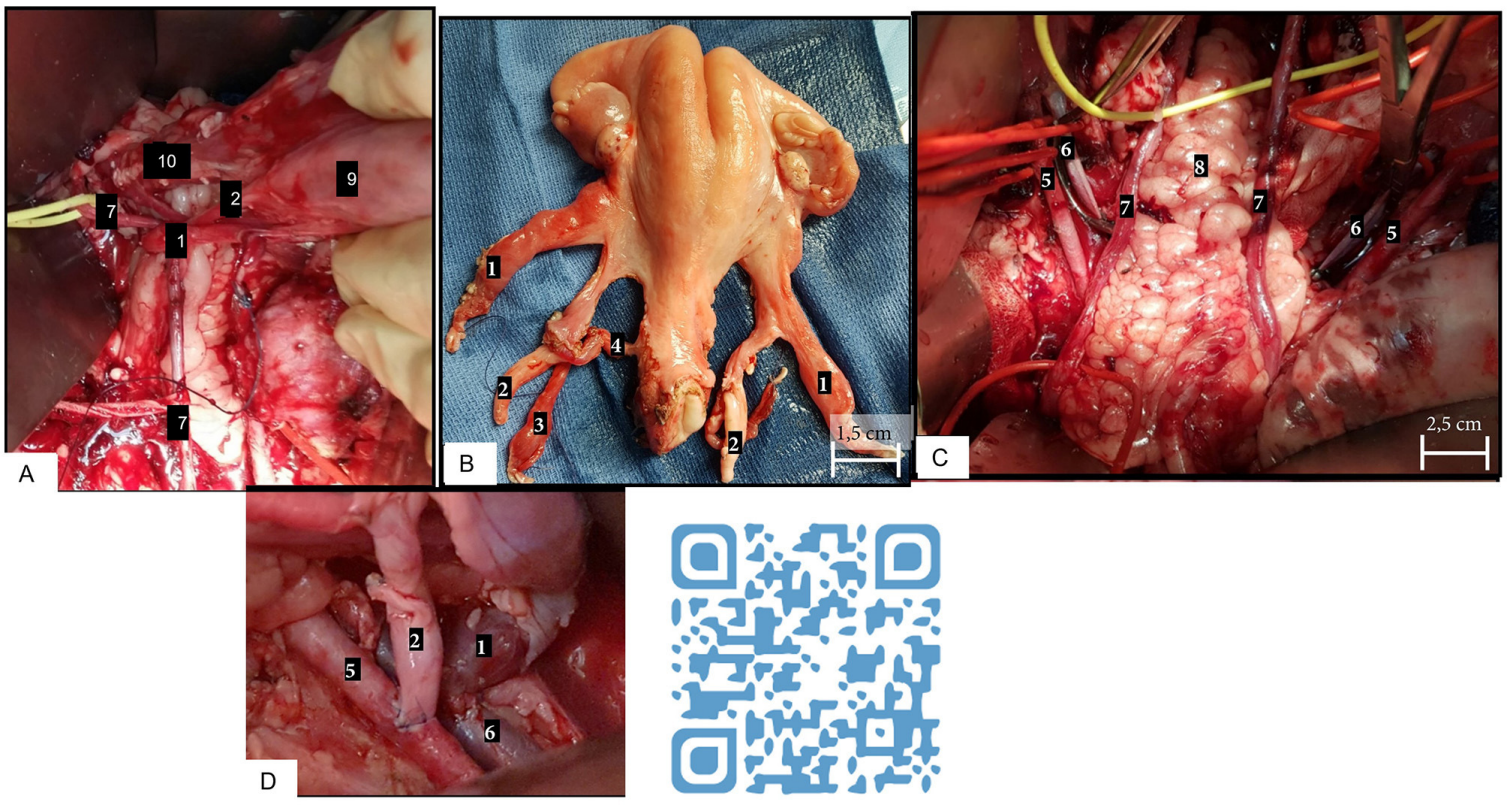
Different steps in UTx in sheep. **(A)** Uterine dissection, lateral view. **(B)** Back table. **(C)** Pelvic view before anastomoses. **(D)** Arterial and venous latero-terminal anastomoses. (1) Utero-ovarian vein; (2) uterine artery; (3) umbilical artery; (4) cervico-uterine arterial branch; (5) external iliac artery; (6) external iliac vein; (7) ureter; (8) rectum; (9) uterine horn; and (10) cervix.

Building a UTx-dedicated team through training on a large-animal model is mandatory before performing the UTx in humans (24). Kisu et al. noticed an 82% success rate when having gained UTx expertise in an animal model and 55.6% without expertise (25). The surgical team should involve both gynecologic surgeons with oncologic skills and transplant surgeons mastering microsurgery and robotics procedures. The ewe autotransplantation training protocol used by the authors is available in the attached video and in Figure 3. The different steps of the surgery, performed after laparotomy, are close to human dissection of uterine arteries and utero-ovarian veins, freeing and retrieval of the uterus, flushing vessels on Back table, and replacing it in an orthotopic position using end-to-side vascular anastomosis with external iliac vessels. One ewe is used as donor and recipient in accordance with the 3R. We found out that in ewes, achieving uterine dissection is possible after five cases and arterial and venous anastomoses are reproducible after seven and nine cases, respectively (17).

## Basic Research

The contribution of different animal models to UTx is summarized in Table 2.

**Table 2:** Uterine transplantation (UTx) studies in animal models.

	**Selection**	**Transplantation techniques**	**Pregnancy**	**Ischemia studies**	**Rejection studies**	**Bioengineering**
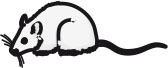		Syngeneic heterotopic transplantation (7, 15, 26) Allogeneic heterotopic transplantation (27–29)	Achieved (7, 30) *In utero* exposure of cyclosporine A (8)	24–48 h cold ischaemic preservation (26)	Rejection patterns (27) Immunosuppression with cyclosporine A (28) Leukocyte subtypes in rejection (31)	
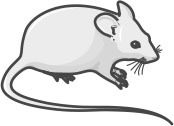		Inbred heterotopic transplantation (32, 33) Allogeneic heterotopic transplantation (34, 35) Syngeneic orthotopic transplantation (13, 36, 37) Allogeneic orthotopic transplantation (38–40)	Achieved in syngeneic (13) and in allogeneic (34) Effect on tacrolimus on offspring (38)	4 h warm ischemia study (37) Remifentanil (41), Melatonin and Glycine (42) or cannabinoid agonist JWH-133 (43) decrease ischemia-reperfusion injury Using Histidine-Tryptophan-Ketoglutarate with acetyl L-carnitine (44) or Custodiol-N (45) for uterus preservation	Immunosuppression (34) Effects of cyclosporine A (39) Effects of tacrolimus (40)	Uterus decellularization (46–48) and recellularizing (49, 50) Bioengineered patches in decellularized uterus permits pregnancy (51) Immune response in transplanted decellularized uterus (36)
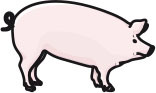		Auto-transplantation (9) Heterotopic (15) and orthotopic (52, 53) allogeneic transplantation		Early reperfusion events (9)	Immunosuppression with tacrolimus and cyclosporine (15)	
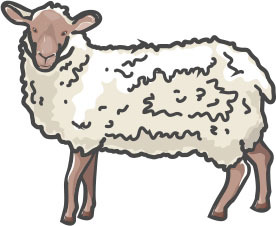	MRI study (54) Multispectral imaging laparoscopy (55) Indocyanine green angiography (56) Angiography (57)	One horn auto-transplantation (58) Orthotopic auto-transplantation (10, 17–19, 55, 59–63) and allotransplantation (64, 83) Laparoscopic auto-transplantation (57)	Achieved in auto-transplantation (64) and allo- transplantation (10)	Early reperfusion events (19, 58, 65) Long cold ischemia evaluation (18, 63) Normothermic *ex vivo* reperfusion model (66)	Immunosuppression with cyclosporine (64)	Uterus decellularization (67) Recellularization in bioactive uterus scaffolds (68)
Baboon
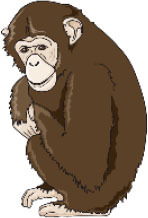		Autotransplantation (69) Autotransplantation with end-to-side or end-to-end anastomoses (21) Autotransplantation with utero-ovarian anastomoses only (30) Autotransplantation using uterine and ovarian pedicles (70) Allogeneic orthotopic transplantation (68, 71)	Achieved in autotransplantation (30)	Long-term reperfusion (69)	Immunosuppression protocol (71) Long-term graft survival (72)	
Macaque
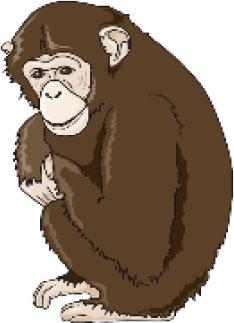	Indocyanine green angiography (73, 74)	Autotransplantation (73, 75) Orthotopic allotransplantation (20, 76)	Achieved in autotransplantation (20) and allotransplantation (77)	Evaluation of warm ischemia (78)	Immunosuppression protocol (79, 80)	

**Figure 4:**
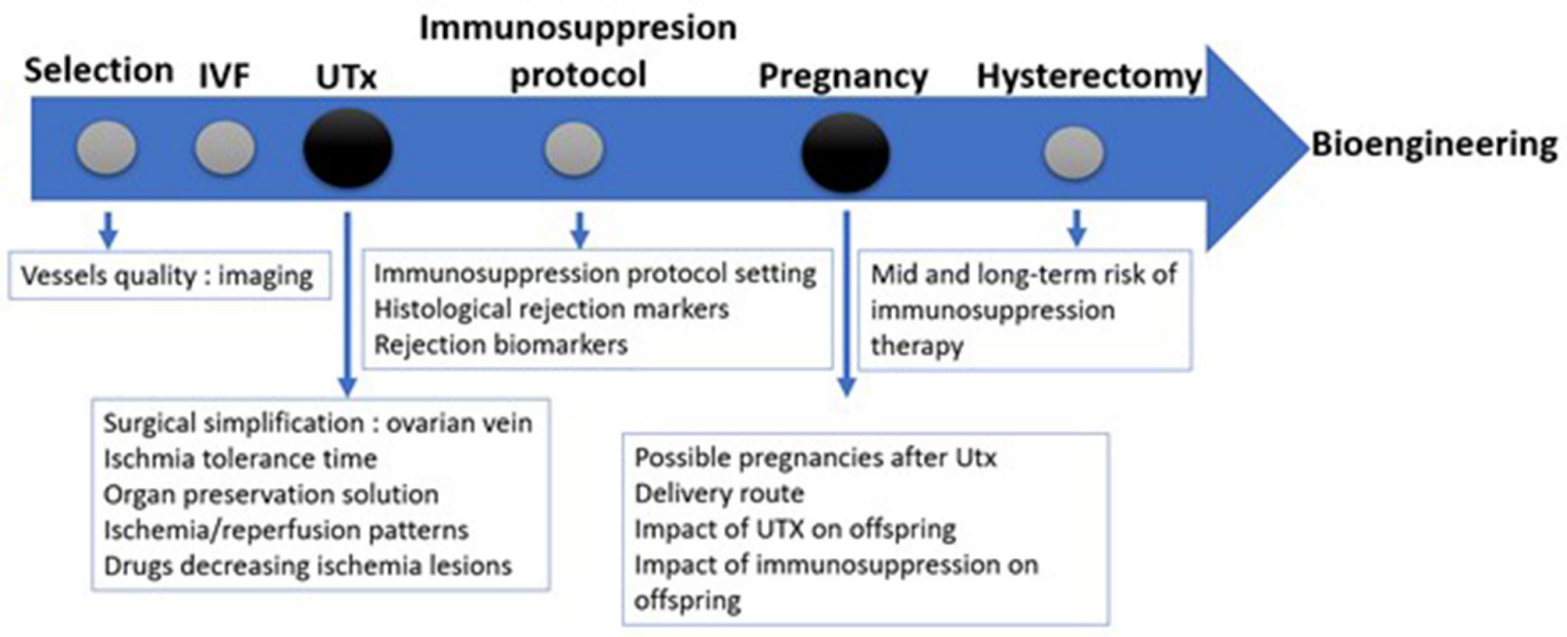
Different steps of UTx and their research domains. IVF: *In vitro* Fertilization.

Different questions at each step of the UTx process were provided by research in different animal models as illustrated in Figure 4. The selection of the candidates (compatible donors and recipients) is the first step. The use of appropriate imaging is mandatory to assess the vessel quality. Before surgery, *in vitro* fertilization (IVF) is performed to obtain enough embryos as fallopian tubes are removed during surgery due to devascularization. Afterward, UTx is realized. Immunosuppression is introduced right away and after assuring that there is no rejection, the embryo can be transferred. Delivery is achieved *via* caesarean section and the uterus is removed after one or two pregnancies.

### Selection: Imaging

Selection of the donor is essential and requires imaging to assess the quality of the graft and its vascularization preoperatively as postoperatively. There are no studies using animal research that looked at CT angiography (CTA), digital subtraction angiography (DSA), and magnetic resonance angiography (MRA) to preoperatively evaluate the “quality” of uterine arteries. Imaging was, however, used in animals to ascertain the quality of the vascularization of the graft after surgery.

A Doppler examination is often used pre- or postoperatively to visualize the blood flow in the transplanted uterus (17). An implantable Doppler Cook-Swartz has been used in ewes (56) to monitor the blood flow postoperatively. This invasive technique used a small ultrasound sensor fixed near the anastomosis to ascertain its permeability.

In addition, MRI was used in ewes, first to define the surgery's feasibility (54) and second (81) to postoperatively analyze the graft. Coupled with ultrasonography, the measurement of uterine size as well as assessment of the presence or absence of graft edema or of an eventual thrombosis of the anastomoses were enabled.

Indocyanine green (ICG) is injected intravenously and identified with a special camera obtaining images of very small vessels intraoperatively. The value of the technique has been proved in the ewe (56) and macaque (73). It can reveal the vascularization of the donor and so show possible anatomical variations. ICG angiography proved that a unique uterine artery on one side in macaque vascularizes both sides of the uterus, cervix, and oviducts (74), while one ovarian artery do not vascularize the cervix and contralateral oviduct. In the ewe (56), ICG angiography enabled evaluation of the permeability of anastomoses and could be repeated when a stenosis is noticed.

Additionally, the multispectral imaging laparoscopy has been used in an autotransplantation ewe model to visualize the organ oxygen saturation during the procedure (82).

In human research, arteriography is the best imaging approach to assess vessels before performing UTx (83). Further studies in animals should be conducted to test other less invasive imaging techniques than arteriography before UTx for evaluating uterine arteries and try to find an appropriate way for imaging veins, as nothing is available to date.

### Surgery

Several surgical techniques were developed to simplify the UTx procedure. The aim of these simplifications was to either reduce the surgical time or test new surgical techniques before implementing them in humans.

In rodents (15, 32), due to the small vessel size, aorta and vena cava or common iliac vessels are collected to perform UTx forcing the sacrifice of the donor. The vessels are anastomosed to the aorta and caudal vena cava of the recipient. This surgical technique enabled much needed basic research. In larger animals, end-to-end anastomoses are performed in a few cases in pigs (9) and in sheep (84). A deceased donor model in sheep was also developed (81), using end–to-side anastomosis of aorta and cava inferior patch to the external iliac vessels.

To use a deceased donor, a technique of perfusion *via* the femoral and/or external iliac artery in the macaques has shown good perfusion of the uterus. This suggests that it is possible to use this perfusion technique in UTx for brain-dead donor instead of classical aorta canulation incompatible with the uterine preservation (85), as successfully demonstrated in humans (86). In sheep (17, 19, 55), macaque (74, 85), and baboon (69), mostly end-to-side anastomoses between uterine vessels and external iliac vessels were performed, which is the surgical technique used in humans. To simplify UTx, some teams used only one-sided anastomoses. Although the graft viability was obtained with this technique, organ perfusion was significantly reduced. A bilateral revascularization of the graft was therefore necessary to allow gestation on a transplanted uterus (20, 87).

In ewes, ovarian veins (17, 19, 55) were used and short-term drainage of the uterus was feasible through a single venous anastomosis (65). A model in macaque showed that using only the ovarian vein compared with the deep uterine vein was less invasive (88). The outcomes in both groups in terms of vascular injuries and uterine function were similar.

This model was translated in humans and showed favorable issues with functional uterus and live birth (89). A uterine autotransplantation in baboons showed good results when using ovarian veins (70). Furthermore, a description of live births in baboons with an angiosome using microsurgically anastomosed utero-ovarian vessels and lacking uterine arteries and veins is a promising research for reducing morbidity of the surgery for donors in humans (30).

### Ischemia-Reperfusion

Ischemic injuries occur when the grafts stopped being longer perfused. There are two different types of ischemia: warm and cold. Both are responsible for uterine injuries on all levels: molecular, cellular, and tissue. The ischemia-reperfusion syndrome can lead to acute graft rejection (90). In ovine model, several markers for ischemia-reperfusion were searched using the histological, immunohistochemistry, and molecular biology approaches (17, 19, 65). The increase in some ischemia markers was due to oxidative stress and inflammation induced by ischemia-reperfusion (65).

Warm ischemia can rapidly induce uterine damages. Histopathological changes due to warmth ischemia were studied in cynomolgus monkeys (78). Warm ischemia can cause permanent damage if it lasts more than 4 h. Within 3 h, there were no histological or functional injuries, and these results that are transposable to the humans. In rats, the injection of Melatonin and Glycine (42), Remifentanil (41), or cannabinoid agonist JWH-133 (43) reduced warm ischemia-reperfusion injuries.

The uterus can tolerate up to 24 h of cold ischemia in mice (26). Grafts were preserved 24 h in a heparinized isotonic saline solution and transplanted successfully. The results showed a good uterine resilience after a long time of cold ischemia. A reperfusion model in ovine (66) was developed to evaluate the tolerance of the uterus for increased cold ischemia times. Uteri were placed for 4–48 h in cold ischemia and reperfused for 48 h in normothermic conditions with a sterile solution which composition was close to blood whereas the temperature and oxygen level can be modified. This showed favorable outcomes with normothermic *ex vivo* reperfusion and allowed to study ischemia-reperfusion markers *ex vivo*.

Several preservation solutions were used in animal models to improve the quality of the grafts. Custodiol-N (45) was better than Custodiol for uterine preservation in rats. Adding acetyl L-carnitine to a Histidine-Tryptophan-Ketoglutarate solution prevented the formation of free radicals (44). Perfadex® (10) containing colloid and dextran 40 was better than saline solution for UTx in sheep. By preventing the interaction of activated neutrophils with the vascular endothelium, it protects microvascularization from inflammatory lesions and prevents edema formation during the graft preservation.

Uterine function is dependent upon ischemia-reperfusion injuries. Further basic research on intra- or postoperative therapies leading to a reduction of these lesions will improve the chances of success of this difficult surgery. The challenge for reducing ischemia is mandatory and, there are two ways to do so: improve surgery and find treatments.

### Graft Rejection

Similar to other kinds of transplantation, the uterus can present acute or chronic rejection. Diagnosis of rejection in transplanted organs is made when its functionality decreases leading to several symptoms and blood markers. Uterus rejection is difficult to diagnose because there are no symptoms or blood test expressing uterine functionality. The classification for acute forms uterine rejection in baboons is illustrated in Table 3 (71), which led to establish the classification that is now used in human UTx.

**Table 3:** Classification of acute uterine rejection in endocervical biopsy samples in baboon (71).

**Grade**	**Rejection**	**Biopsy findings**
0	No	Normal morphology
1	Mild	Mild diffuse mixed inflammatory cell infiltrate (mainly lymphocytes). Occasional epithelial apoptotic bodies, focal distribution. Surface epithelium intact. No necrosis
2	Moderate	Moderate, diffuse mixed inflammatory cell infiltrate (mainly lymphocytes). Increased amount of epithelial apoptotic bodies: Reduced thickness surface epithelium, possible focal erosion. No necrosis
3	Severe	Significant, diffuse and aggregate, mixed inflammatory cell infiltrate (mainly lymphocytes; neutrophils and eosinophils may be present). Frequent apoptotic bodies. Epithelial erosions, focal to total. Focal necrosis
4	Total necrosis	Necrotic tissue only

Non-invasive rejection markers need to be found to facilitate the follow-up of transplanted patients.

### Immunosuppressive Protocol

Immunosuppressive drugs are mandatory to avoid organ rejection in every allotransplantation. After achieving syngeneic UTx, allotransplantation comes with immunological challenges.

The first allogeneic transplantations (27) were achieved in mice and showed the need for immunosuppressive therapy. Without treatment, the inflammation was visible 2 days after transplantation. After 10–15 days, inflammation was maximal, leading to necrotic changes in the graft 28 days after transplantation. Thereafter, cyclosporine A was used in the same model as immunosuppressive therapy. This drug (28) delayed but did not stop uterine graft rejection in the mid and long course. Moreover, high doses of cyclosporine A (13) reduced the embryo implantation rates and increased the fetal mortality rates. One live birth in an ovine model was obtained with cyclosporine A treatment (91).

Fujimycin was used in a rat model and several full-term gestations were obtained with this treatment. There was, however, a significant failure rate requiring further research (34). A triple immunosuppressive therapy (fujimycin, cyclosporine, and methylprednisolone) enabled a 50% long-term survival rate in a mini-pig model (15). The phylogenetic distance of these models from the human species, however, did not allow the extrapolation of the obtained results.

The first immunosuppressive protocol used in a non-human primate allogeneic UTx was published in 2013 (71). Fujimycin, mycophenolate, and corticoids were combined after proving that a monotherapy of fujimycin was insufficient. In macaques, a protocol using antithymocyte globulin, cyclosporine, tacrolimus, mycophenolate mofetil, and methylprednisolone was used but found to be not enough effective (79). When rituximab was added to this protocol, it enabled a good immunological control (77).

These studies need to be considered with precaution because the human immune response can differ from non-human primates. Research in organ transplantation demonstrated the efficiency of some immunosuppressive protocols in humans whereas they were only partially effective in monkeys (92). More research on immunosuppressive protocols is thus needed to bring the right balance between immunosuppression and complications.

### Pregnancy

Uterine transplantation makes sense if the transplanted organ can support the development of the embryo and allow its development to term. From the transplanted uterus, this implies the resumption of endometrial receptivity and a vascularization compatible with placental development and adequate vascularization to allow placentation and proper exchanges with the fetus.

In mice (7), gestation was obtained after embryo transfer in a syngenetic heterotopic model. The offspring of transplanted dams had the same development, growth or fertility as compared with control. A syngenetic orthotopic UTx model in rats (13) has also enabled gestation. There was more miscarriage in the transplanted animals: out of eight gestations, only one led to a successful vaginal birth, with numerous dystocia. This suggested the need of caesarean section for delivery after UTx. In rats, two male rats were born after UTx and their development was normal. Gestation was achieved in an orthotopic allotransplantation in rats (34, 38) with a higher miscarriage rate in transplanted animals. There were discordant data about the number of offspring at birth. In the first study (34), the number of raccoons was lower in the transplanted group, but in the second (38), the opposite was seen. Delivery occurred by caesarean section. The male raccoons from the transplanted uteri were larger than controls but there was no difference in female raccoons.

The first gestation in a UTx sheep model (13) occurred after autotransplantation. The gestation rate was similar in the transplanted group compared with controls. Three gestations resulted in the birth of two lambs, born by C-section, in good health. Uterine torsion was diagnosed in the third gestation, demonstrating the necessity of a good uterine ligament fixation.

There were gestations using an ovine model of allotransplantation in 2011 (91). Twelve ewes underwent transplantation and five could be selected for gestation. Frozen embryos were transferred in two ewes, one of which failed to be pregnant and the other had an extrauterine pregnancy. Fresh embryos were transferred to three other ewes, of which one did not get pregnant, one had a miscarriage, and one went to term. The lamb was born by the caesarean section 10 days before term. The analysis of its vital parameters showed no difference with those published in other studies on preterm lambs.

Gestation was first achieved in 2012 in an auto-transplantation model (20) in the macaque. An emergency caesarean section was performed because of placental abruption. The baby macaque showed signs of fetal distress and was not reanimated for ethical reasons. Normal fetal development was observed at post-mortem examination. Pregnancy was achieved lately in the macaque uterus allotransplantation (77). Gestation was attempted for three macaques in allotransplantation but only one could give birth after 3 miscarriages. A caesarean section was performed, and the offspring showed good development.

Normal pregnancies are possible in all animal models, but transplanted uterus bring more miscarriages. There are cases of fetal distress, uterine torsion, extrauterine pregnancy, and the need to practice a caesarian section to give birth.

More research is needed nowadays to identify the factors in UTx that are associated with uncomplicated pregnancies and livebirths. Among these factors, the impact of immunosuppressive treatments and UTx itself on long-term development in offspring is still poorly investigated and further studies are needed. Small models, such as rodents can be the prefect model because gestation time is very short and so long-term follow-up is possible.

## Discussion and Perspectives

Lately bioengineering has been given new perspectives for uterine replacement (93). Indeed, it should be possible to create an artificial organ using a three-dimensional scaffold that would be cellularized with stem cells. This would make immunosuppressive therapy unnecessary.

Experiments on uterus decellularization were performed in rats (36). A patch of collagen and bone marrow-derived mesenchymal stem cells was inserted in a severely injured uterus. This patch has allowed an increased ability of endometrium, uterine muscle, and microvasculature regeneration (94). A matrix has been partially decellularized and has been transplanted (51). This transplanted uterus gave birth, but placentation occurred only on the cellularized patches.

Lately (95), a pregnancy was carried out using a rabbit model with an autologous cell-seeded engineered uterus. These uteri allowed fetal development and livebirth.

## Conclusion

The use of animal models has played an essential role in the implementation of UTx in humans. Surgical training using large animal models is mandatory for any team wishing to initiate an UTx project. Research using animal models aiming at simplifying this complex new procedure is still necessary using animals even if 3R must be respected as much as possible. Hopefully, bioengineering will come up with an artificial uterus model, which will 1 day bring us the perfect mean for freeing us from animal models.

## Author Contributions

AF-I, MC, and JD wrote the manuscript. AF-I and JD collected the data. NC, SC, RC, FV, VG, LG, CR, HT, OS, DdZ, PC-P, and J-MA read and edited the manuscript. All authors approved the submitted version.

## Conflict of Interest

The authors declare that the research was conducted in the absence of any commercial or financial relationships that could be construed as a potential conflict of interest.

## Publisher's Note

All claims expressed in this article are solely those of the authors and do not necessarily represent those of their affiliated organizations, or those of the publisher, the editors and the reviewers. Any product that may be evaluated in this article, or claim that may be made by its manufacturer, is not guaranteed or endorsed by the publisher.

## References

[1] Johannesson L Järvholm S. Uterus transplantation: current progress and future prospects. Int J Womens Health. (2016) 8:43. 10.2147/IJWH.S75635 26917976 PMC4751897

[2] Brännström M Johannesson L Bokström H Kvarnström N Mölne J Dahm-Kähler P. Livebirth after uterus transplantation. Lancet. (2015) 385:607–16. 10.1016/S0140-6736(14)61728-1 25301505

[3] Brännström M Belfort MA Ayoubi JM. Uterus transplantation worldwide: clinical activities and outcomes. Curr Opin Organ Transplant. (2021) 26:616–26. 10.1097/MOT.0000000000000936 34636769

[4] Moore FD. Ethical problems special to surgery. Arch Surg. (2000) 135:14. 10.1001/archsurg.135.1.14 10636340

[5] McCulloch P Altman DG Campbell WB Flum DaR Glasziou P Marshall JC. No surgical innovation without evaluation: the IDEAL recommendations. Lancet. (2009) 374:1105–12. 10.1016/S0140-6736(09)61116-8 19782876

[6] Milliez J. Uterine transplantation. Int J Gynecol Obstet. (2009) 106:270. 10.1016/j.ijgo.2009.03.045 19501356

[7] Racho El-Akouri R Kurlberg G Brännström M. Successful uterine transplantation in the mouse: pregnancy and post-natal development of offspring. Hum Reprod. (2003) 18:2018–23. 10.1093/humrep/deg396 14507815

[8] Groth K Brännström M Mölne J Wranning CA. Cyclosporine A exposure during pregnancy in mice: effects on reproductive performance in mothers and offspring. Hum Reprod. (2010) 25:697–704. 10.1093/humrep/dep470 20085916

[9] Wranning CA El-Akouri RR Lundmark C Dahm-Kahler P Molne J Enskog A. Auto-transplantation of the uterus in the domestic pig (Sus scrofa): Surgical technique and early reperfusion events. J Obstet Gynaecol Res. (2006) 32:358–67. 10.1111/j.1447-0756.2006.00426.x 16882260

[10] Wranning CA Marcickiewicz J Enskog A Dahm-Kahler P Hanafy A Brannstrom M. Fertility after autologous ovine uterine-tubal-ovarian transplantation by vascular anastomosis to the external iliac vessels. Hum Reprod. (2010) 25:1973–9. 10.1093/humrep/deq130 20519245

[11] MacArthur Clark J. The 3Rs in research: a contemporary approach to replacement, reduction and refinement. Br J Nutr. (2018) 120:S1–7. 10.1017/S0007114517002227 29081302

[12] Racho El-Akouri R Kurlberg G Dindelegan G Mölne J Wallin A Brännström M. Heterotopic uterine transplantation by vascular anastomosis in the mouse. J Endocrinol. (2002) 174:157–66. 10.1677/joe.0.1740157 12176655

[13] Wranning CA Akhi SN Diaz-Garcia C Brannstrom M. Pregnancy after syngeneic uterus transplantation and spontaneous mating in the rat. Hum Reprod. (2011) 26:553–8. 10.1093/humrep/deq358 21159686

[14] Sieunarine K Zakaria FBP Boyle DCM Corless DJ Noakes DE Lindsay I. Possibilities for fertility restoration: a new surgical technique. Int Surg. (2005) 90:249–56. 10.1016/j.fertnstert.2005.07.1229 16625941

[15] Avison DL Defaria W Tryphonopoulos P Tekin A Attia GR Takahashi H. Heterotopic uterus transplantation in a swine model. Transplantation. (2009) 88:465–9. 10.1097/TP.0b013e3181b07666 19696628

[16] Dion L Le Lous M Nyangoh Timoh K Levêque J Arnaud A Henri-Malbert C. Single bilateral ovarian venous return in uterine transplant: validation in an orthotopic auto-transplant model in the Yucatan minipig. J Gynecol Obstet Hum Reprod. (2021) 50:102059. 10.1016/j.jogoh.2021.102059 33421624

[17] Favre-Inhofer A Carbonnel M Revaux A Sandra O Mougenot V Bosc R. Critical steps for initiating an animal uterine transplantation model in sheep: experience from a case series. Int J Surg. (2018) 60:245–51. 10.1016/j.ijsu.2018.11.017 30481612

[18] Andraus W Ejzenberg D Santos R Mendes L Arantes R Baracat E. Sheep model for uterine transplantation: the best option before starting a human program. Clinics. (2017) 72:178–82. 10.6061/clinics/2017(03)08 28355364 PMC5348579

[19] Dahm-Kähler P Wranning C Lundmark C Enskog A Mölne J Marcickiewicz J. Transplantation of the uterus in sheep: methodology and early reperfusion events. J Obstet Gynaecol Res. (2008) 34:784–93. 10.1111/j.1447-0756.2008.00854.x 18834335

[20] Mihara M Kisu I Hara H Iida T Araki J Shim T. Uterine autotransplantation in cynomolgus macaques: the first case of pregnancy and delivery. Hum Reprod. (2012) 27:2332–40. 10.1093/humrep/des169 22647448

[21] Johannesson L Enskog A Dahm-Kähler P Hanafy A Chai D Mwenda JM. Uterus transplantation in a non-human primate: long-term follow-up after autologous transplantation. Hum Reprod. (2012) 27:1640–8. 10.1093/humrep/des093 22454459

[22] Brännström M Johannesson L Dahm-Kähler P Enskog A Mölne J Kvarnström N. First clinical uterus transplantation trial: a six-month report. Fertil Steril. (2014) 101:1228–36. 10.1016/j.fertnstert.2014.02.024 24582522

[23] Brännström M Dahm-Kähler P Kvarnström N. Robotic-assisted surgery in live-donor uterus transplantation. Fertil Steril. (2018) 109:256–7. 10.1016/j.fertnstert.2017.12.007 29395094

[24] Brännström M Diaz-Garcia C Hanafy A Olausson M Tzakis A. Uterus transplantation: animal research and human possibilities. Fertil Steril. (2012) 97:1269–76. 10.1016/j.fertnstert.2012.04.001 22542990

[25] Kisu I Kato Y Obara H Matsubara K Matoba Y Banno K. Emerging problems in uterus transplantation. BJOG. (2018) 125:1352–6. 10.1111/1471-0528.15230 29869370

[26] Racho El-Akouri R Wranning CA Mölne J Kurlberg G Brännström M. Pregnancy in transplanted mouse uterus after long-term cold ischaemic preservation. Hum Reprod. (2003) 18:2024–30. 10.1093/humrep/deg395 14507816

[27] El-Akouri RR Mölne J Groth K Kurlberg G Brännström M. Rejection patterns in allogeneic uterus transplantation in the mouse. Hum Reprod. (2005) 21:436–42. 10.1093/humrep/dei349 16253976

[28] Wranning CA El-Akouri RR Groth K Mölne J Parra A Brännström M. Rejection of the transplanted uterus is suppressed by cyclosporine A in a semi-allogeneic mouse model. Hum Reprod. (2007) 22:372–9. 10.1093/humrep/del410 17062584

[29] Yu S Xie B Zhang L Song Y Yang Y An K. Live birth after cervical ectopic uterus transplantation in mice. Am J Transplant. (2020) 20:2226–33. 10.1111/ajt.15831 32092213

[30] Beran B Arnolds K Shockley M Rivas K Medina M Escobar PF. Livebirth and utero-placental insufficiency in Papio hamadryas baboons with uterus angiosome perfused by bilateral utero-ovarian microsurgical anastomoses alone. Hum Reprod. (2017) 32:1819–26. 10.1093/humrep/dex242 28854716

[31] Groth K Akouri R Wranning CA Molne J Brannstrom M. Rejection of allogenic uterus transplant in the mouse: time-dependent and site-specific infiltration of leukocyte subtypes. Hum Reprod. (2009) 24:2746–54. 10.1093/humrep/dep248 19617209

[32] Wranning CA Akhi SN Kurlberg G Brännström M. Uterus transplantation in the rat: Model development, surgical learning and morphological evaluation of healing. Acta Obstet Gynecol Scand. (2008) 87:1239–47. 10.1080/00016340802484966 18951268

[33] Jiga LP Lupu CM Zoica BS Ionac M. Experimental model of heterotopic uterus transplantation in the laboratory rat. Microsurgery. 10.1002/micr.10134. (2003);23:246–50. 10.1002/micr.10134 12833326

[34] Díaz-García C Akhi SN Wallin A Pellicer A Brännström M. First report on fertility after allogeneic uterus transplantation. Acta Obstet Gynecol Scand. (2010) 89:1491–4. 10.3109/00016349.2010.520688 20879912

[35] Padma AM Alsheikh AB Song MJ Akouri R Akyürek LM Oltean M. Immune response after allogeneic transplantation of decellularized uterine scaffolds in the rat. Biomed Mater. (2021) 16. 10.1088/1748-605X/abfdfe 33946053

[36] Padma AM Alshaikh AB Song MJ Akouri R Oltean M Brännström M. Decellularization protocol-dependent damage-associated molecular patterns in rat uterus scaffolds differentially affect the immune response after transplantation. J Tissue Eng Regen Med. (2021) 15:674–85. 10.1002/term.3217 33991074

[37] Díaz-García C Akhi SN Martínez-Varea A Brännström M. The effect of warm ischemia at uterus transplantation in a rat model. Acta Obstet Gynecol Scand. (2013) 92:152–9. 10.1111/aogs.12027 23061896

[38] Díaz-García C Johannesson L Shao R Bilig H Brännström M. Pregnancy after allogeneic uterus transplantation in the rat: perinatal outcome and growth trajectory. Fertil Steril. (2014) 102:1545–52.e1. 10.1016/j.fertnstert.2014.09.010 25439799

[39] Groth K Akhi SN Mölne J Wranning CA Brännström M. Effects of immunosuppression by cyclosporine A on allogenic uterine transplant in the rat. Eur J Obstet Gynecol Reprod Biol. (2012) 163:97–103. 10.1016/j.ejogrb.2012.03.026 22502817

[40] Akhi SN Diaz-Garcia C El-Akouri RR Wranning CA Mölne J Brännström M. Uterine rejection after allogeneic uterus transplantation in the rat is effectively suppressed by tacrolimus. Fertil Steril. (2013) 99:862–70. 10.1016/j.fertnstert.2012.11.002 23218920

[41] Atalay YO Aktas S Sahin S Kucukodaci Z Ozakpinar OB. Remifentanil protects uterus against ischemia-reperfusion injury in rats. Acta Cir Bras. (2015) 30:756–61. 10.1590/S0102-865020150110000006 26647795

[42] Zitkute V Kvietkauskas M Maskoliunaite V Leber B Ramasauskaite D Strupas K. Melatonin and glycine reduce uterus ischemia/reperfusion injury in a rat model of warm ischemia. Int J Mol Sci. (2021) 22:8373. 10.3390/ijms22168373 34445081 PMC8394613

[43] Aydogan Kirmizi D Baser E Doganyigit Z. The activation of cannabinoid type-2 receptor with JWH-133 protects uterine ischemia/reperfusion-induced damage. Pharmacology. (2021) 106:106–13. 10.1159/000511457 33105141

[44] Ugurlu T Ozogul C Saribas GS Gurgen SG Akyol SN Kartal B. The effect of antioxidants on angiogenesis in uterine transplantation. J Obstet Gynaecol. (2018) 38:382–7. 10.1080/01443615.2017.1316250 29385880

[45] Zitkute V Kvietkauskas M Maskoliunaite V Leber B Ramasauskaite D Strupas K. Custodiol-N is superior to custodiol ® solution in experimental rat uterus preservation. Int J Mol Sci. (2020) 21:1–10. 10.3390/ijms21218015 33126511 PMC7662817

[46] Hellström M El-Akouri RR Sihlbom C Olsson B Lengqvist J Bäckdahl H. Towards the development of a bioengineered uterus: comparison of different protocols for rat uterus decellularization. Acta Biomater. (2014) 10:5034–42. 10.1016/j.actbio.2014.08.018 25169258

[47] Santoso EG Yoshida K Hirota Y Aizawa M Yoshino O Kishida A. Application of detergents or high hydrostatic pressure as decellularization processes in uterine tissues and their subsequent effects on *in vivo* uterine regeneration in murine models. PLoS ONE. (2014) 9:e103201. 10.1371/journal.pone.0103201 25057942 PMC4109986

[48] Miyazaki K Maruyama T. Partial regeneration and reconstruction of the rat uterus through recellularization of a decellularized uterine matrix. Biomaterials. (2014) 35:8791–800. 10.1016/j.biomaterials.2014.06.052 25043501

[49] Li X Wang Y Ma R Liu X Song B Duan Y. Reconstruction of functional uterine tissues through recellularizing the decellularized rat uterine scaffolds by MSCs *in vivo* and *in vitro*. Biomed Mater. (2021) 16 035023. 10.1088/1748-605X/abd116 33660616

[50] Padma AM Tiemann TT Alshaikh AB Akouri R Song MJ Hellström M. Protocols for rat uterus isolation and decellularization: applications for uterus tissue engineering and 3D cell culturing. Methods Mol Biol. (2018) 1577:161–75. 10.1007/7651_2017_60 28776178

[51] Hellström M Moreno-Moya JM Bandstein S Bom E Akouri RR Miyazaki K. Bioengineered uterine tissue supports pregnancy in a rat model. Fertil Steril. (2016) 106:487–96.e1. 10.1016/j.fertnstert.2016.03.048 27068301

[52] Oliveira E de Tavares KA da S Gomes MTV Salzedas-Netto AA Sartori MGF Castro RA. Description and evaluation of experimental models for uterine transplantation in pigs. Einstein. (2017) 15:481–5. 10.1590/s1679-45082017ao4066 29267429 PMC5875164

[53] Zhang X Liu J Wu Q Liu Z Yan Z. Uterus Allo-Transplantation in a Swine Model: Long-Term Graft Survival and Reproductive Function. Med Sci Monit. (2018) 24:8422–9. 10.12659/MSM.913051 30465552 PMC6262904

[54] Gauthier T Marquet P Kanoun D Maubon A Piver P Couquet C. Pelvic magnetic resonance imaging in the ewe: a model for experimental gynecologic research. J Obstet Gynaecol Res. (2014) 40:133–8. 10.1111/jog.12141 24033802

[55] Saso S Petts G Thum M-Y Corless D Boyd M Noakes D. Achieving uterine auto-transplantation in a sheep model using iliac vessel anastomosis: a short-term viability study. Acta Obstet Gynecol Scand. (2015) 94:245–52. 10.1111/aogs.12550 25421489

[56] Kengelbach-Weigand A Lotz L Schmid R Lang W Beckmann MW Hoffmann I. Intra- and postoperative blood flow monitoring in a sheep model of uterus transplantation. In vivo. (2019) 33:325–36. 10.21873/invivo.11478 30804109 PMC6506297

[57] Sánchez-Margallo FM Moreno-Naranjo B Pérez-López M del M Abellán E Domínguez-Arroyo JA Mijares J. Laparoscopic uterine graft procurement and surgical autotransplantation in ovine model. Sci Rep. (2019) 9:8095. 10.1038/s41598-019-44528-1 31147586 PMC6543039

[58] Wranning CA Dahm-KP Nilsson UA Enskog A Br M. Transplantation of the uterus in the sheep: oxidative stress and reperfusion injury after short-time cold storage. J Obstet Gynaecol Res. (2008) 34:784–93. 10.1111/.1447-0756.2008.00854.x 17904131

[59] Tricard J Ponsonnard S Tholance Y Mesturoux L Lachatre D Couquet C. Uterus tolerance to extended cold ischemic storage after auto-transplantation in ewes. Eur J Obstet Gynecol Reprod Biol. (2017) 214:162–7. 10.1016/j.ejogrb.2017.05.013 28535402

[60] Solomonov E Marcus Braun N Siman-Tov Y Ben-Shachar I. Team preparation for human uterus transplantation: Autologous transplantation in sheep model. Clin Transplant. (2017) 31:e13137. 10.1111/ctr.13137 29032587

[61] Maraschio MA Larcher JMS Alcaraz A Giordano E Reimondez S Luján O. Uterus transplantation in a sheep model: novel surgical technique with long-term survival and uterus vitality. First case series in Argentina. JBRA Assist Reprod. (2021) 25 557–62. 10.5935/1518-0557.20210035 34463443 PMC8489826

[62] Arantes RM Nacif LS Pinheiro RS Rocha-Santos V de Martino RB Waisberg DR. Novel technique in a sheep model of uterine transplantation. Transplant Proc. (2020) 52:1399–401. 10.1016/j.transproceed.2020.02.040 32276834

[63] Tardieu A Chazelas P Faye PA Favreau F Nadal-Desbarats L Sallée C. Changes in the metabolic composition of storage solution with prolonged cold ischemia of the uterus. J Assist Reprod Genet. (2019) 36:1169–78. 10.1007/s10815-019-01477-y 31079269 PMC6603114

[64] Ramirez ER Ramirez DK Pillari VT Vasquez H Ramirez HA. Modified uterine transplant procedure in the sheep model. J Minim Invasive Gynecol. (2008) 15:311–4. 10.1016/j.jmig.2008.01.014 18439503

[65] Carbonnel M Cornet N Revaux A Favre-Inhofer A Galio L Raliou M. Analysis of blood parameters and molecular endometrial markers during early reperfusion in two ovine models of uterus transplantation. PLoS ONE. (2021) 16:e0251474. 10.1371/journal.pone.0251474 34003831 PMC8130915

[66] Padma AM Truong M Jar-Allah T Song MJ Oltean M Brännström M. The development of an extended normothermic *ex vivo* reperfusion model of the sheep uterus to evaluate organ quality after cold ischemia in relation to uterus transplantation. Acta Obstet Gynecol Scand. (2019) 98:1127–38. 10.1111/aogs.13617 30932168

[67] Tiemann TT Padma AM Sehic E Bäckdahl H Oltean M Song MJ. Towards uterus tissue engineering: a comparative study of sheep uterus decellularisation. Mol Hum Reprod. (2020) 26:167–78. 10.1093/molehr/gaaa009 31980817 PMC7103571

[68] Padma AM Carrière L Krokström Karlsson F Sehic E Bandstein S Tiemann TT. Towards a bioengineered uterus: bioactive sheep uterus scaffolds are effectively recellularized by enzymatic preconditioning. NPJ Regen Med. (2021) 6:26. 10.1038/s41536-021-00136-0 34021161 PMC8140118

[69] Enskog A Johannesson L Chai DC Dahm-Kahler P Marcickiewicz J Nyachieo A. Uterus transplantation in the baboon: methodology and long-term function after auto-transplantation. Hum Reprod. (2010) 25:1980–7. 10.1093/humrep/deq109 20519250

[70] Han MN Ramirez H Ruvalcaba L Contreras JL Nyachieo A Ramirez E. Uterine autotransplantation in the nonhuman primate with preservation of the uterine and ovarian vascular pedicles. Reprod Sci. (2018) 26:1329–35. 10.1177/1933719118765976 29576000

[71] Johannesson L Enskog A Molne J Diaz-Garcia C Hanafy A Dahm-Kahler P. Preclinical report on allogeneic uterus transplantation in non-human primates. Hum Reprod. (2013) 28:189–98. 10.1093/humrep/des381 23108346

[72] Tryphonopoulos P Tzakis AG Tekin A Johannesson L Rivas K Morales PR. Allogeneic uterus transplantation in baboons: surgical technique and challenges to long-term graft survival. Transplantation. (2014) 98:e51–6. 10.1097/TP.0000000000000322 25171537

[73] Mihara M Kisu I Hara H Iida T Yamamoto T Araki J. Uterus autotransplantation in cynomolgus macaques: intraoperative evaluation of uterine blood flow using indocyanine green. Hum Reprod. (2011) 26:3019–27. 10.1093/humrep/der276 21896548

[74] Kisu I Banno K Mihara M Lin LY Tsuji K Yanokura M. Indocyanine green fluorescence imaging for evaluation of uterine blood flow in cynomolgus macaque. PLoS ONE. (2012) 7:e35124. 10.1371/journal.pone.0035124 22606213 PMC3335048

[75] Kisu I Mihara M Banno K Hara H Yamamoto T Araki J. A new surgical technique of uterine auto-transplantation in cynomolgus monkey: preliminary report about two cases. Arch Gynecol Obstet. (2012) 285:129–37. 10.1007/s00404-011-1901-2 21475964 PMC3249183

[76] Obara H Kisu I Kato Y Yamada Y Matsubara K Emoto K. Surgical technique for allogeneic uterus transplantation in macaques. Sci Rep. (2016) 6:35989. 10.1038/srep35989 27786258 PMC5081522

[77] Kisu I Kato Y Masugi Y Ishigaki H Yamada Y Matsubara K. First successful delivery after uterus transplantation in mhc-defined cynomolgus macaques. J Clin Med. (2020) 9:1–23. 10.3390/jcm9113694 33213083 PMC7698480

[78] Adachi M Kisu I Nagai T Emoto K Banno K Umene K. Evaluation of allowable time and histopathological changes in warm ischemia of the uterus in cynomolgus monkey as a model for uterus transplantation. Acta Obstet Gynecol Scand. (2016) 95:991–8. 10.1111/aogs.12943 27329637

[79] Kisu I Ishigaki H Emoto K Kato Y Yamada Y Matsubara K. Long-term outcome and rejection after allogeneic uterus transplantation in cynomolgus macaques. J Clin Med. (2019) 8:1572. 10.3390/jcm8101572 31581534 PMC6833021

[80] Kisu I Mihara M Banno K Hara H Masugi Y Araki J. Uterus allotransplantation in cynomolgus macaque: a preliminary experience with non-human primate models. J Obstet Gynaecol Res. (2014) 40:907–18. 10.1111/jog.12302 24612366

[81] Gauthier T Bertin F Fourcade L Maubon A Saint Marcoux F Piver P. Uterine allotransplantation in ewes using an aortocava patch. Hum Reprod. (2011) 26:3028–36. 10.1093/humrep/der288 21896546

[82] Clancy NT Saso S Stoyanov D Sauvage V Corless DJ Boyd M. Multispectral imaging of organ viability during uterine transplantation surgery in rabbits and sheep. J Biomed Opt. (2016) 21:106006. 10.1117/1.JBO.21.10.106006 27786342

[83] Leonhardt H Thilander-Klang A Båth J Johannesson M Kvarnström N Dahm-Kähler P. Imaging evaluation of uterine arteries in potential living donors for uterus transplantation: a comparative study of MRA, CTA, and DSA. Eur Radiol. (2021). 10.1007/s00330-021-08350-6 PMC8921132 34767069

[84] Wei L Xue T Yang H Zhao GY Zhang G Lu ZH. Modified uterine allotransplantation and immunosuppression procedure in the sheep model. PLoS ONE. (2013) 8:e81300. 10.1371/journal.pone.0081300 24278415 PMC3838404

[85] Kisu I Kato Y Yamada Y Matsubara K Obara H Emoto K. Organ perfusion for uterus transplantation in non-human primates with assumed procurement of a uterus from a brain-dead donor. Transplant Proc. (2016) 48:1266–9. 10.1016/j.transproceed.2015.12.105 27320600

[86] Chmel R Pastor Z Novackova M Matecha J Cekal M Fronek J. Clinical pregnancy after deceased donor uterus transplantation: Lessons learned and future perspectives. J Obstet Gynaecol Res. (2019) 45:1458–65. 10.1111/jog.13992 31062518

[87] Arvin J Ashrafi M Lotfi S Dadvand Meshgin Shahr A Schomann E Scheerer K. Experimental uterus transplant in various models: review of surgical techniques. Exp Clin Transplant. (2018) 16:119–26. 29621960

[88] Kisu I Banno K Mihara M Hara H Umene K Adachi M. A surgical technique using the ovarian vein in non-human primate models of potential living-donor surgery of uterus transplantation. Acta Obstet Gynecol Scand. (2015) 94:942–8. 10.1111/aogs.12701 26095999

[89] Testa G McKenna GJ Gunby RT Anthony T Koon EC Warren AM. First live birth after uterus transplantation in the United States. Am J Transplant. (2018) 18:1270–4. 10.1111/ajt.14737 29575738

[90] Desrois M Caus T Dalmasso C Lan C Cozzone P Bernard M. Expression of the three nitric oxide synthase isoforms and nitric oxide level in the rat heart during cold storage and blood reperfusion. Cell Mol Biol. (2009) 55(Supp. 1):1208–14. 20018145

[91] Ramirez ER Ramirez Nessetti DK Nessetti MBR Khatamee M Wolfson MR Shaffer TH. Pregnancy and outcome of uterine allotransplantation and assisted reproduction in sheep. J Minim Invasive Gynecol. (2011) 18:238–45. 10.1016/j.jmig.2010.11.006 21354071

[92] Brännström M Wranning CA Altchek A. Experimental uterus transplantation. Hum Reprod Update. (2010) 16:329–45. 10.1093/humupd/dmp049 19897849

[93] Johannesson L Dahm-Kähler P Eklind S Brännström M. The future of human uterus transplantation. Womens Health. (2014) 10:455–67. 10.2217/WHE.14.22 25259905

[94] Ding L Li X Sun H Su J Lin N Péault B. Transplantation of bone marrow mesenchymal stem cells on collagen scaffolds for the functional regeneration of injured rat uterus. Biomaterials. (2014) 35:4888–900. 10.1016/j.biomaterials.2014.02.046 24680661

[95] Magalhaes RS Williams JK Yoo JJ Atala A. A tissue-engineered uterus supports live births in rabbits. Nat Biotechnol. (2020) 38:1280–7. 10.1038/s41587-020-0547-7 32601434 PMC7641977

